# Sensory substitution in bilateral vestibular a-reflexic patients

**DOI:** 10.14814/phy2.12385

**Published:** 2015-05-13

**Authors:** Bart B G T Alberts, Luc P J Selen, Wim I M Verhagen, W Pieter Medendorp

**Affiliations:** 1 Radboud University Nijmegen, Donders Institute for Brain, Cognition and BehaviourMontessorilaan 3, 6525HR, Nijmegen, the Netherlands; 2 Neurology, Canisius Wilhelmina HospitalWeg Door Jonkerbos 100, 6532 SZ, Nijmegen, the Netherlands

**Keywords:** Bilateral vestibular a-reflexia, internal models, multisensory integration, spatial orientation, verticality perception

## Abstract

Patients with bilateral vestibular loss have balance problems in darkness, but maintain spatial orientation rather effectively in the light. It has been suggested that these patients compensate for vestibular cues by relying on extravestibular signals, including visual and somatosensory cues, and integrating them with internal beliefs. How this integration comes about is unknown, but recent literature suggests the healthy brain remaps the various signals into a task-dependent reference frame, thereby weighting them according to their reliability. In this paper, we examined this account in six patients with bilateral vestibular a-reflexia, and compared them to six age-matched healthy controls. Subjects had to report the orientation of their body relative to a reference orientation or the orientation of a flashed luminous line relative to the gravitational vertical, by means of a two-alternative-forced-choice response. We tested both groups psychometrically in upright position (0°) and 90° sideways roll tilt. Perception of body tilt was unbiased in both patients and controls. Response variability, which was larger for 90° tilt, did not differ between groups, indicating that body somatosensory cues have tilt-dependent uncertainty. Perception of the visual vertical was unbiased when upright, but showed systematic undercompensation at 90° tilt. Variability, which was larger for 90° tilt than upright, did not differ between patients and controls. Our results suggest that extravestibular signals substitute for vestibular input in patients’ perception of spatial orientation. This is in line with the current status of rehabilitation programs in acute vestibular patients, targeting at recognizing body somatosensory signals as a reliable replacement for vestibular loss.

## Introduction

Patients with vestibular function loss have a deteriorated sense of spatial orientation, leading to balance problems in darkness, especially on irregular surfaces. In the light, however, this lack of spatial orientation often remains unnoticed (Verhagen et al. 2000). This suggests that spatial orientation is not only governed by vestibular signals, but also depends on extravestibular sensory signals. In support, previous studies in healthy subjects have shown that multiple sensory systems can provide graviceptive signals (Mittelstaedt 1992, 1995, 1996; Lackner and DiZio 2005; Angelaki and Cullen 2008; Carriot et al. 2011). The integration of these extravestibular signals, together with internal beliefs about likely body orientations (Eggert 1998; De Vrijer et al. 2008, 2009), could compensate for the lack of vestibular information in bilateral patients.

**Figure 1:**
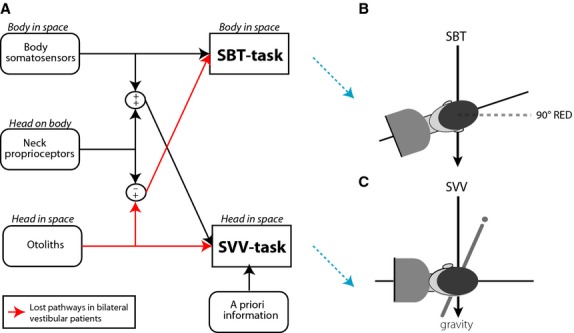
(A) Schematic representation of the multisensory integration model by Clemens et al. (2011) In the SBT task, body somatosensory signals provide direct information about orientation of body orientation in space, whereas the otoliths provide indirect information about the body orientation in space by taking into account the head-on-body information provided by neck proprioception. Similarly, in the SVV task otoliths provide direct information while body somatosensory signals combined with neck proprioceptors provide indirect information about head orientation in space. Both direct and indirect pathways are optimally combined for best performance on the tasks. Note that in the SVV task prior information about our head-in-space influences these pathways. Secondly, to compute the SVV, the brain also uses estimates of eye-in-head orientation (ocular counterroll) and line orientation on the retina (not shown here). The red arrows indicate information pathways that are lost in bilateral vestibular patients. (B) SBT task: subject has to indicate whether body orientation is clockwise (CW) or counterclockwise (CCW) of a certain reference orientation (dashed line) (C) SVV task: subjects are rotated to either upright or 90° RED and have to indicate whether a luminous line flashed in front of them is oriented clockwise (CW) or counterclockwise (CCW) of gravity.

How vestibular and extravestibular signals interact in spatial orientation is difficult to assess because they cannot be measured in isolation. Recently, Clemens et al. (2011) proposed a novel computational approach to estimate the contributions of the various sensory systems in spatial orientation of healthy subjects by testing both the perception of body tilt (SBT, subjective body tilt) and of the visual vertical (SVV, subjective visual vertical). While both tasks require integration of the same sensory signals, their different task constraints impose different interactions between the signals (Fig.1A). For example, in SBT body somatosensory signals provide direct information about body orientation in space, whereas otolith information needs to be combined with head-on-body information from neck proprioceptors to provide an estimate of body orientation in space. Similarly, in SVV otoliths provide direct head-in-space information, whereas body somatosensory signals combined with neck proprioceptors provide indirect information. These two pathways are integrated together with internal beliefs to provide an estimate of head-in-space orientation.

Using an inverse probabilistic analysis, Clemens et al. (2011) showed that healthy subjects rely mostly on vestibular signals when being upright, reverting to an increased reliance on body somatosensory signals at larger tilts, attributed to the increased vestibular noise. An untested prediction of their Bayesian multisensory integration model is that when vestibular information is lost (i.e., bilateral vestibular patients), biases should become larger in SVV tasks, and response variability should be increased, but constant over the whole tilt range, in both SVV and SBT tasks compared to healthy controls.

While there are numerous studies on SBT and SVV in unilateral vestibular patients (reviewed by Pérennou et al. (2014)), only few studies tested bilateral vestibular patients (Bisdorff et al. 1996; Bronstein et al. 1996; Bronstein 1999; Bringoux 2002; Lopez et al. 2007). However, none of these studies tested SVV and SBT within the same patient group, at multiple tilt angles, or provided quantitative values of bias and variability.

The present study is the first to assess psychometrically both the SBT and SVV in six patients with bilateral vestibular function loss due to DFNA9 (DeaFNess Autosomal 9). Their vestibular loss arises from an acidophilic mucopolysaccharide deposit, identified in the cochlea and macula, that causes strangulation of the nerve endings (Huygen et al. 1989, 1991; Verhagen et al. 2000; Cremers et al., 2005; Robertson et al., 2006)

Our results suggest these patients use alternative sensory pathways to compensate for vestibular loss, amplifying signals related to neck proprioception and body somatosensation. In computational terms, our results can be explained by assuming body somatosensory noise to be multiplicative and not additive, as in the original model by Clemens et al. (2011).

## Materials and Methods

### Subjects

Six naive patients (four female, two male, age 62 ± 10 years.) with vestibular a-reflexia due to a hereditary progressive vestibulo-cochlear dysfunction caused by a COCH gene mutation (DFNA9) participated in the experiment (Verhagen et al. 2000). Complete loss of vestibular function was confirmed in several clinical tests (see Table1). Otolith function was tested by video recording of eye movements during an ocular counterroll (OCR) task. Patients showed no ocular counterroll when the head was statically tilted on the trunk to 25°. In three patients, additional myogenic potentials due to bone vibration of the head were recorded by surface EMG electrodes underneath the eyes (oVEMP) and at the sternocleidomastoid muscles (cVEMP, both air-conducted and bone-conducted). Loss of both utricular (oVEMP) and saccular (cVEMP) function was confirmed by the absence of any myogenic potentials. Absence of nystagmus during 4 cm eccentric off axis constant speed rotation further confirmed these observations. In addition to otolith testing, various clinical semicircular canal tests were performed. First, caloric tests, performed with 30 sec irrigation of 150–200 cm^3^ water at 30°C and 44°C, did not induce reactive eye movements. Second, velocity step tests, with rotational velocities of 90°/sec (all patients) and 250°/sec (in four patients), showed no postrotary nystagmus responses, all indicating canal loss. There was no response during acceleration either. In addition to testing the vestibular apparatus directly, both previous literature and the current study have shown an increase in optokinetic response gain (Huygen et al. 1989; Huygen and Verhagen 2011) and cervical ocular reflex gain (Huygen et al. 1991), both indicative of compensatory mechanisms for total vestibular loss.

**Table 1:** Clinical tests performed to show vestibular a-reflexia

	Patient 1	Patient 2	Patient 3	Patient 4	Patient 5	Patient 6
Otolith tests						
4 cm off-axis rotation	No nystagmus	–	No nystagmus	–	–	No nystagmus
oVEMP^1^	No response	–	No response	No response	–	–
cVEMP^2^	No response	–	No response	No response	–	–
OCR video^3^	No OCR	No OCR	No OCR	No OCR	No OCR	No OCR
Canal tests						
VOR (90°) step test^4^	No postrotary nystagmus	No postrotary nystagmus	No postrotary nystagmus	No postrotary nystagmus	No postrotary nystagmus	No postrotary nystagmus
VOR (250°) step test^4^	No postrotary nystagmus	–	No postrotary nystagmus	–	No postrotary nystagmus	No postrotary nystagmus
Caloric test (30 and 44°C)	No reactive eye movements	No reactive eye movements	No reactive eye movements	No reactive eye movements	No reactive eye movements	No reactive eye movements
Other tests						
OKR gain^5^	↑	↑	↑	↑	–	↑
COR gain^6^	↑	↑	↑	–	–	–

1 Ocular Vestibular Evoked Myogenic Potential measured underneath the eyes.

2 Cervical Vestibular Evoked Myogenic Potential measured at the sternocleidomastoid muscle (air-conducted and bone-conducted).

3 Video recording of eye movements during Ocular Counterroll.

4 Vestibular Ocular Reflex initiated by velocity step tests.

5 Measuring the response gain of the eyes during optokinetic stimulation.

6 Measuring the response gain of the eyes during body under head rotation.

–: test was not performed in the patient.

Although vestibular function is completely lost, some patients still had a small amount of remaining auditory function; typically vestibular loss precedes total hearing loss in DFNA9 (Bischoff et al. 2005). Auditory function was supported by hearing aids or restored by cochlear implants. One patient suffered from diabetes mellitus with a mild polyneuropathy; the other patients had no additional neurological abnormalities. All had normal or corrected to normal vision.

Six naive, age-matched control subjects (four male, two female, age 61 ± 11 years) were also tested. Integrity of the vestibular system in control subjects was not clinically tested, but subjects reported to be free of any known vestibular or other neurological disorders and had normal or corrected to normal vision.

Both patients and controls gave written informed consent to the guidelines of the local ethics committee. Prior to the experiment, subjects were carefully instructed about the tasks and performed a few practice trials in the light. Subjects never received feedback about their performance, not even in the practice trials. Each subject participated in three experimental sessions, yielding about 2 h recording time.

### Setup

A computer-controlled vestibular chair was used to rotate subjects in roll with an angular resolution of 0.04° (see Clemens et al. (2011)). The subject's body was tightly fixated using a five-point seat belt and adjustable shoulder and hip supports. Velcro straps restrained both legs and feet, and a padded helmet firmly fixated the head in a natural upright position for looking straight ahead. Subject-specific seat adjustments ensured comfort seating and that the naso-occipital axis coincided with the roll axis of the chair. Experiments took place in complete darkness.

### Experiments

All patients and controls were tested in both the subjective body tilt task (SBT) and the subjective visual vertical task (SVV), following the psychophysical procedures described in Clemens et al. (2011) (Fig.1). We limited our measurements to only two tilt angles: upright and 90° right-ear-down (RED). We chose these reference angles because they should reveal the largest difference between patients and controls (Clemens et al. 2011). Furthermore, we optimized the number of trials needed for a veridical psychometric analysis, yielding 100 and 110 trials for the SBT and SVV task, respectively. With both adjustments, we ensured that Clemens et al. (2011) methods could still be applied while at the same time keeping the experiment viable for our patients. The two experimental tasks were as follows.

#### SBT

We applied the method of constant stimuli, using a set of 10 equidistant body-tilt angles, centered on 0° and 90° RED, separated by intervals of 3° and 4°, respectively. Each experimental run started in the upright position with the room lights on. After the lights were turned off, subjects were first rotated at a constant angular velocity of 30°/sec to a random detour angle, outside of the test angle range, where they remained for 1 sec. Detour angles were chosen randomly from a range 20–30° clockwise (CW) and counterclockwise (CCW) from the reference angle. The chair then moved to the test angle using a very slow and noisy profile, defined by the sum of a ramp (0.2–4°/sec) °/sec) and filtered Gaussian white noise (bandwidth, 0.5 Hz; RMS amplitude, 3.4°). We introduced the noisy profile to deter reliance on sensed changes in tilt position that had occurred since the previous trial (see also Fig.2 of Clemens et al. 2011). Immediately after arrival at the test angle, a beep signal prompted the subject to indicate whether body orientation was CW or CCW from the instructed reference orientation (upright or 90° RED) using a toggle switch. The subject was then rotated at constant velocity of 30°/sec to a new randomly drawn detour angle, and the above procedure was repeated. Each run comprising 10 test angles lasted approximately 4 min, after which the subject was rotated back to upright, and room lights were turned on. Between runs, there was a 30 sec rest interval. Subjects performed 10 runs for each reference orientation, yielding 100 trials. The two reference orientations (0° and 90° RED) were tested in separate sessions of about 45 min each.

**Figure 2:**
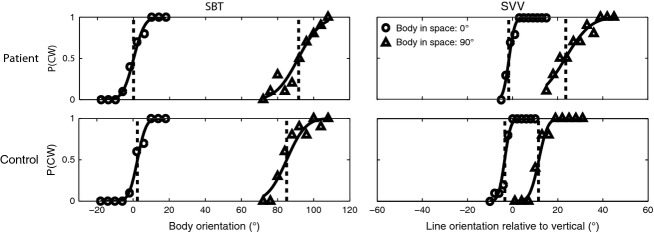
SBT and SVV performance for a typical control and patient. SBT: proportion of clockwise responses P(CW) is plotted against body orientation centered on the two reference angles (0° or 90° RED). SVV: proportion of clockwise responses P(CW) is plotted against line orientation with respect to the vertical when the body is either upright or at 90° RED.

#### SVV

The SVV was also tested in upright and 90° RED position, using the method of constant stimuli. An adjustable luminous line (angular subtend 20°), polarized with a bright dot at one end, was mounted in front of the subject such that the line's rotation axis coincided with the nasal occipital axis of the subject. In each experimental run, the subject was rotated from upright to the chosen test angle (upright or 90° RED) at a constant angular velocity of 30°/sec. After a 30 sec waiting period that allowed canal effects to subside, the luminous line was flashed for 20 msec and subjects indicated whether its orientation was CW or CCW from their perceived direction of gravity. All 11 line orientations were presented around a coarse estimate of the SVV accuracy in pseudorandom order in each run. After each run, the subject was rotated back to upright, and room lights were turned on. One run lasted about 1 min, in which subjects remained at the same roll tilted position for about 45 sec. Based on previous work (Clemens et al. 2011), line orientation intervals were chosen to be 2° and 3° for the upright and 90° RED positions, respectively. For each condition, 10 experimental runs were conducted, yielding a total of 110 responses for each test angle. Both conditions were randomly intermixed across the 20 experimental runs and collected in a single 30-min session.

### Data analysis

CW tilt angles of the body and the luminous line were defined positive. A cumulative Gaussian, including a lapse rate, was fitted to the psychometric data using maximum likelihood (Wichmann and Hill 2001).

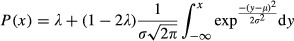


*P*(*x*) is the probability of a CW response, given a body orientation (SBT) or line orientation (SVV). The orientation *x* for which *P*(*x*) becomes 0.5, that is, *x* = *μ*, is the orientation where subjects perceive their body orientation equal to the reference orientation or where they perceive the luminous line oriented along the gravitational vertical. We took *μ* as a measure for accuracy of the percept; a bias exists if *μ *≠ 0. The width of the curve, *σ*^2^, serves as a measure of the variability in the percept. For each subject, a single lapse rate *λ*, restricted to small values (*λ *< 0.15), accounted for stimulus-independent errors in all conditions.

### Statistical analysis

All analyses were performed offline using Matlab 2012a (The MathWorks, Inc., Natick, MA) and SPSS 19 (IBM Corp, Armonk, NY). We compared the effect of group (patient vs. control) and orientation (upright vs. 90° RED) on SBT and SVV performance using a two-way univariate analysis of variance with subject as a random factor. Interaction effects were post hoc analyzed using a Bonferroni-corrected paired sample *t*-test. All statistical tests were performed at the 0.05 level (*P* < 0.05).

## Results

Figure2 shows the performance of a single vestibular patient and a typical control subject in both the SBT (left column) and SVV task (right column). Each panel demonstrates how the fraction of CW-responses changes as a function of body orientation (in the SBT) or line orientation relative to the perceived vertical (in the SVV), for the 0° (circles) and 90° RED orientation (triangles).

The SBT data show that the patient is unbiased at both reference angles. Response variability increases slightly for the larger tilt angle. Both the bias and response variability look quite similar to those of the control subject, whose performance resembles previous literature (Mittelstaedt 1983; Mast and Jarchow 1996; Jarchow and Mast 1999; Van Beuzekom and Van Gisbergen 2000; Van Beuzekom et al. 2001; Kaptein and Van Gisbergen 2004; De Vrijer et al. 2008; Vingerhoets et al. 2008; Clemens et al. 2011). We fit psychometric curves to these data (see Methods) to obtain quantitative measures for the bias and response variability. As indicated by the vertical dashed line, the point of subjective equality is near veridical in both patient and control. Response variability is captured by the width of the curve. The increased width of the psychometric curve for the 90° tilt angle indeed captures the observation that response variability is larger for the 90° reference orientation than at upright.

The right-hand panels of Fig.2 illustrate the psychometric data and subsequent fits for the response data of the SVV task. Both the patient and control subject are unbiased in the upright conditions; response variability seems smaller than in the SBT task. The fits confirm both observations. For the 90° tilt angle, there is a clear systematic bias, as if both patient and control underestimate their tilt angle. The patient further shows a larger bias than the control subject. Performance at this angle is also marked by increased response variability compared to the upright position, as in the SBT task. The patient's variability is also slightly larger than that of the control subject, whose response pattern matches with previous reports (Bisdorff et al. 1996). The fitted psychometric curves indicate that patient and control perform generally similar, with slight differences at 90° tilt.

### No significant differences between vestibular patients and healthy controls in SBT task

**Figure 3:**
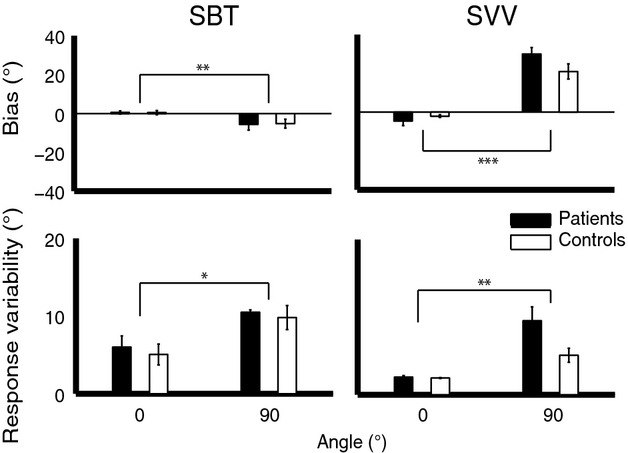
Mean bias and response variability at the upright and 90° roll tilt in both the SBT and SVV task. Error bars denote the standard error across subjects. * indicates *P* < 0.05, **P < 0.01, and ***P < 0.001.

Figure3 depicts the summary statistics (mean and SE) across the six patients and six control subjects, generalizing the observations described in Fig.2. We subjected bias and response variability values, as obtained from the psychometric fits, to a univariate ANOVA with factors angle (0° and 90°) and group (patients and controls). For the SBT, there was no difference in bias between patients and controls (*F*_(1,5)_ = 0.005, *P* = 0.95). A significant effect of angle was observed (*F*_(1,5)_ = 20.11, *P* = 0.006), which can be explained by the small (patients: −5.7 ± 7.0, controls: −5.3 ± 5.6), but systematic, underestimation at 90°. There was no interaction effect between group and angle (*F*_(1,5)_ = 0.009, *P* = 0.93) Response variability was higher for the RED compared to the upright condition (*F*_(1,5)_ = 16.11, *P* = 0.01), but no effect of group (*F*_(1,5)_ = 0.20, *P* = 0.68) or interaction between group and angle (*F*_(1,5)_ = 0.036, *P* = 0.86) was observed.

### SVV bias and variability at 90° tilt tend to be larger for patients

For the SVV, the biases showed a significant effect of angle (*F*_(1,4.999)_ = 99.31, *P* = 0.0002) but no effect of group (*F*_(1,3.99)_ = 0.65, *P* = 0.47). However, group did have a significant interaction effect with angle (*F*_(1,3)_ = 40.86, *P* = 0.008). Although Fig.3 indicates a trend toward a larger bias for patients relative to controls, statistical testing showed no significant group effect at 0° (*t*_(1,4)_ = −1.71, *P* = 0.16) or 90° tilt (*t*_(1,3)_ = 2.87, *P* = 0.064). As in the SBT task, response variability was higher for the 90° tilt condition (*F*_(1,4.992)_ = 32.35, *P* = 0.002), but there was no effect of group (*F*_(1,3.957)_ = 6.08, *P* = 0.070) or an interaction between the two factors (*F*_(1,3)_ = 4.85, *P* = 0.12). One should note that the difference in bias between groups at 90° tilt and the difference in variability between groups are close to statistical significance.

## Discussion

We compared the biases and response variability in patients with vestibular a-reflexia to that of age-matched controls when estimating body orientation relative to a reference angle (SBT) and line orientation relative to the gravitational vertical (SVV). Regarding the first (SBT), both groups were virtually unbiased in indicating the direction of roll tilt relative to upright and at 90° RED. Furthermore, both groups showed a significant increase in response variability with larger tilt angle. For the SVV task, both groups were unbiased at upright and showed a substantial deviation at 90° RED. This effect was slightly more pronounced in the patients as indicated by a significant interaction effect. Response variability increased with larger tilt angle for both groups. In both SBT and SVV variability, there were no significant differences between groups. So, despite the absence of any vestibular input, patients’ performance differed only marginally from the controls. We will now first compare our results to previous work and then discuss their further implications for the model and rehabilitation.

### The presented results are consistent with previous (clinical) studies

Studies on the perception of spatial orientation in bilateral vestibular patients have so far only been conducted in either SBT or SVV tasks, often for a single roll angle only and using nonpsychometric estimates of bias and variability. From these studies, Bisdorff et al. (1996) and Bringoux (2002) estimated the SBT at upright and showed that patients are as accurate as healthy controls. Bisdorff et al. (1996) further showed that patients have an increased variability over healthy controls when at upright. This is contrary to our results, but could be explained by their measure of uncertainty that is based on threshold detection and not response variability.

Other clinical studies reported increased SVV biases for bilateral vestibular patients over healthy controls, in both upright and tilted conditions (Bronstein et al. 1996; Bronstein 1999; Guerraz et al. 2001; Lopez et al. 2007). Close scrutiny of these studies, however, indicates that the SVV was always measured in the presence of optokinetic stimulation. The optokinetically induced effect is likely to be stronger in vestibular patients because they rely more on visual information than healthy controls (Huygen et al. 1989; Huygen and Verhagen 2011).

Recently, Valko et al. (2012) tested dynamic tilt perception in patients with total vestibular loss, showing motion discrimination thresholds during roll rotation about twice as high as healthy controls. While this indicates an important role of vestibular cues in dynamic tilt perception, caution should be taken when extrapolating their results to static tilt perception, for which contribution of other extravestibular cues might be weighted more heavily.

### Implications for multisensory integration

It is clear that a representation of gravity is required to determine our body orientation in space and the orientation of objects in the external world. Because of physics reasons, sensory systems are often ambiguous as to this representation. For example, according to Einstein's equivalence principle, accelerations due to translation or gravity cannot be distinguished.

The brain may rely on an internal model of this physics (Merfeld et al. 1999), but why then is there a discrepancy in performance between SBT and SVV at 90° tilt? Subjects know that they are tilted 90° relative to gravity, but show substantial biases in the perception of vertical. This intriguing paradox was first described by Mittelstaedt (1983), followed by many other studies (Mittelstaedt 1983; Mast and Jarchow 1996; Jarchow and Mast 1999; Van Beuzekom and Van Gisbergen 2000; Van Beuzekom et al. 2001; Kaptein and Van Gisbergen 2004; De Vrijer et al. 2008; Vingerhoets et al. 2008; Clemens et al. 2011). Mittelstaedt proposed that the visual vertical is determined by a weighted combination of a sensory head-tilt signal and a head-fixed reference, which mitigates the different gains of both otolith components because the utricle contains more hair cells than the saccule (Mittelstaedt 1983).

In contrast, the statistical model by Clemens et al. (2011) assumes that otolith signals become more noisy, not biased, with tilt increase, presumably due to the nonuniform distribution of hair cells. In this model, the otolith signal is combined with the prior assumption that the head is usually upright to yield a more stable, but biased, percept of the visual vertical than can be derived from the otolith signal alone.

How can this model explain the errors and variability in our patients, which lack otolith function? Following the Clemens framework of Fig.1A, SBT responses are based on the direct pathway only, since information along the indirect pathway has been cut off (red arrows). This suggests that the response variability in the SBT task, presented in Fig.3, reflects the noise properties of the somatosensory sense, transformed to a behavioral reference frame. A further inference is that the noise in the somatosensory system depends on tilt angle and therefore is multiplicative, not additive.

In the SVV task, the sense of body orientation needs to be combined with visual information about the luminous line to estimate the visual vertical. Figure3 shows a trend that patients are more biased than controls at 90° RED roll tilt, confirming the idea that spatial orientation is multisensory and an optimal integration of all senses is used to estimate the line orientation (Mittelstaedt 1992, 1995; MacNeilage et al. 2007; Vingerhoets et al. 2008; Tarnutzer et al. 2009a,b).

### Neurophysiological implications

Signals from the otoliths are sent to the vestibular nuclei, the first central stage of neural processing. Neurons in the vestibular nuclei are, however, not purely vestibular; they also receive visual, motor, and somatosensory information (Dickman and Angelaki 2004; Angelaki and Cullen 2008; Sadeghi et al. 2012; Carriot et al. 2013). This multisensory convergence in the vestibular nucleus has led to the belief that it may be involved in higher level cognitive functions like spatial orientation (for review see Angelaki and Cullen (2008).

Our results indicate that an extravestibular tilt-dependent noise source is involved in the estimation of the SBT and SVV. This source could in fact be multifaceted, arising from cutaneous receptors that sense the change in the distribution of pressure on the skin, from muscle tension that is increased and/or from the putative visceral graviceptors in the trunk (Mittelstaedt 1995). Although we are not aware of any direct evidence that the noise of these sensors increases with tilt angle, one might argue that they share the same decoding process as the otoliths (Clark et al. 2015) and other sensors (Sober and Körding 2012); when the signal increases the sensors are still accurate, but less precise.

If this holds, a similar SVV bias should be seen when vestibular cues are intact but somatosensory cues are lacking, as in somatosensory patients (Clemens et al. 2011). Indeed, studies attenuating somatosensory signals (water immersion, whole body casts) and lesion studies confirm this hypothesis, showing that without body somatosensory signals, response bias and variability increase with tilt angle in the SVV task (Anastasopoulos et al. 1999; Trousselard et al. 2003, 2004; Barra et al. 2010).

### Spatial orientation through sensory substitution

It has been argued that two distinctive mechanisms can account for recovery of functioning after sensory loss: sensory restitution and sensory substitution (Curthoys 2000). Applied to the vestibular system, restitution would mean the (partial) recovery of vestibular sense due to the use of other senses, whereas substitution would mean that other senses take over the function of the vestibular sense. Our patients show no response to vestibular stimulation tasks years after the vestibular a-reflexia was identified (Table1), suggesting that they have adapted to rely on the remaining, nonvestibular signals and that sensory substitution applies to the spatial orientation performance of our patients. This is confirmed by monkey studies showing sensory substitution at the first stage of vestibular processing where highly multimodal inputs are received (Sadeghi et al. 2012; Cullen 2014; Jamali et al. 2014).

The findings of our study support the current status of vestibular rehabilitation programs tailored toward recognizing body somatosensory signals as a reliable replacement of the vestibular loss in acute vestibular patients (Hillier and McDonnell 2011; McCall and Yates 2011; Deveze et al. 2014). However, our DFNA9 patients lost their vestibular function over the course of years and as a result have probably gradually learned to rely on extravestibular signals to substitute vestibular loss.

### Limitations of the present study

Although the present approach and subsequent data set is one of the most extensive studies in a patient group with full bilateral vestibular deficits, a number of limitations can still be listed. First, patients with bilateral vestibular loss, who satisfied the inclusion criterion are not very frequent. Although all our clinical tests showed that patients have full vestibular loss, it cannot be excluded that some vestibular function remained. If so, this could never explain the very similar performance of patients and controls, upon which we based the arguments for sensory substitution. That said, we tested only six patients, and six respective controls, which should be taken into account in the interpretation of some of the statistical trends. The present study was also limited to measurements of only two tilt angles: upright and 90⁰ right-ear-down tilt. It should be realized that the present 2AFC approach, which is the most quantitative method available, is also very time consuming. Especially measuring the SBT, which is the basis of our claim of signal-dependent noise of body sensors, takes a substantial amount of time (45 min per tilt angle). Testing more tilt angles would have been desired, but was too taxing for our patient population. We like to emphasize that measuring intermediate tilt angles would not change our main conclusion that there is a tilt dependence on the noise properties of the body sensors.

## Conflict of Interest

None declared.
